# An In-Depth Characterization of the Major Psoriasis Susceptibility Locus Identifies Candidate Susceptibility Alleles within an *HLA-C* Enhancer Element

**DOI:** 10.1371/journal.pone.0071690

**Published:** 2013-08-19

**Authors:** Alex Clop, Anna Bertoni, Sarah L. Spain, Michael A. Simpson, Venu Pullabhatla, Raul Tonda, Christian Hundhausen, Paola Di Meglio, Pieter De Jong, Adrian C. Hayday, Frank O. Nestle, Jonathan N. Barker, Robert J. A. Bell, Francesca Capon, Richard C. Trembath

**Affiliations:** 1 Division of Genetics and Molecular Medicine, King’s College London, London, United Kingdom; 2 Centre for Research in Agricultural Genomics (CRAG), Campus Universitat Autònoma de Barcelona, Cerdanyola del Valles, Spain; 3 BACPAC Resources Centre, Children’s Hospital Oakland, Oakland, California, United States of America; 4 Division of Immunology, Infection & Inflammatory Diseases, King’s College London, London, United Kingdom; 5 Department of Neurosurgery, Helen Diller Family Comprehensive Cancer Center, University of California San Francisco, San Francisco, California, United States of America; 6 Queen Mary University of London, Barts and the London School of Medicine and Dentistry, London, United Kingdom; Tor Vergata University of Rome, Italy

## Abstract

Psoriasis is an immune-mediated skin disorder that is inherited as a complex genetic trait. Although genome-wide association scans (GWAS) have identified 36 disease susceptibility regions, more than 50% of the genetic variance can be attributed to a single Major Histocompatibility Complex (MHC) locus, known as *PSORS1.* Genetic studies indicate that *HLA-C* is the strongest *PSORS1* candidate gene, since markers tagging HLA-Cw*0602 consistently generate the most significant association signals in GWAS. However, it is unclear whether HLA-Cw*0602 is itself the causal *PSORS1* allele, especially as the role of SNPs that may affect its expression has not been investigated. Here, we have undertaken an in-depth molecular characterization of the *PSORS1* interval, with a view to identifying regulatory variants that may contribute to disease susceptibility. By analysing high-density SNP data, we refined *PSORS1* to a 179 kb region encompassing *HLA-C* and the neighbouring *HCG27* pseudogene. We compared multiple MHC sequences spanning this refined locus and identified 144 candidate susceptibility variants, which are unique to chromosomes bearing HLA-Cw*0602. In parallel, we investigated the epigenetic profile of the critical *PSORS1* interval and uncovered three enhancer elements likely to be active in T lymphocytes. Finally we showed that nine candidate susceptibility SNPs map within a *HLA-C* enhancer and that three of these variants co-localise with binding sites for immune-related transcription factors. These data indicate that SNPs affecting HLA-Cw*0602 expression are likely to contribute to psoriasis susceptibility and highlight the importance of integrating multiple experimental approaches in the investigation of complex genomic regions such as the MHC.

## Introduction

Psoriasis vulgaris is a chronic inflammatory skin disorder, caused by keratinocyte hyper-proliferation, angiogenesis, and infiltration of immune cells into the dermis and the epidermis [1]. The genetic basis of the disease is well documented by a large body of family- and population-based studies, which have convincingly demonstrated a complex mode of inheritance for this condition [2].

Although genome-wide association scans (GWAS) have identified more than 30 disease susceptibility intervals, up to 50% of the genetic variance is accounted for by a single locus (*PSORS1)* [3], which consistently generates odds ratios >2.5 [4]–[6]. This region spans approximately 250 kb within the Major Histocompatibility Complex (MHC) and encompasses nine protein-coding genes [7]. Disease associated alleles are specifically found within *CDSN,* which encodes a keratinocyte structural protein, *CCHCR1,* which regulates EGFR-STAT3 signalling and *HLA-C,* which codes for a class I MHC molecule [8]–[10]. Although all three proteins contribute to biological processes that are relevant to disease pathogenesis, *HLA-C* is widely considered the most likely candidate gene for the *PSORS1* locus, as the association with HLA-Cw*0602 is more significant than that observed with any other marker [5], [6]. The functional relevance of this observation, however, remains unclear, as no -Cw6 specific antigen or interacting protein has yet been identified. At the same time, the existence of regulatory polymorphisms co-segregating with HLA-Cw*0602 cannot be excluded. In fact our group has demonstrated that SNPs residing within the *HLA-C* minimal promoter cause the differential expression of various –Cw alleles, including HLA-Cw*0602 [11].

Here, we have undertaken a systematic analysis of the *PSORS1* interval, with the aim of identifying disease associated variants that have the potential to impact on *HLA-C* expression. By combining the results of high-resolution genetic profiling with an experimental annotation of regulatory sites, we have identified a restricted number of candidate susceptibility alleles mapping to an *HLA-C* enhancer element.

## Results

### High-resolution Analysis of Linkage Disequilibrium Conservation Refines the *PSORS1* Interval to a 179 kb Region

**Figure 1:**
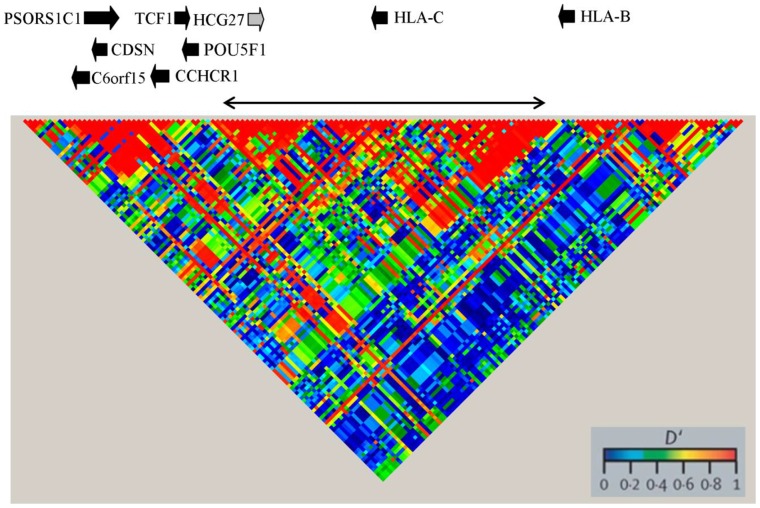
High-resolution analysis of the *PSORS1* interval reveals multiple regions of LD conservation. LD decay was measured using the D’ index, which was derived based on the genotypes of 4,803 UK controls. Markers occurring with a minor allele frequency (MAF) <0.1 were excluded from the analysis, as variation at these loci is likely to be the result of recent mutational events. The plot documents the analysis of 147 SNPs, randomly selected from the 373 variants that had a MAF >0.1 (see Methods for details). The top panel shows the position of *PSORS1* genes (the *HCG27* pseudogene is shaded in grey), with the region of LD conservation around *HLA-C* highlighted by a double arrow.

We sought to refine the boundaries of the *PSORS1* interval, by dissecting the pattern of linkage disequilibrium (LD) conservation around the *HLA-C* locus. We exploited the availability of the high-density SNP data generated by the ImmunoChip Consortium, which genotyped six-hundred and fifteen *PSORS1* markers in 4,803 UK controls [3]. By analysing this extended study population, we noted that the *PSORS1* locus encompasses several LD bocks (Figure 1). We also observed that *HLA-C* lies within a 179 kb region of LD conservation, which excludes all other protein coding genes. This refined susceptibility interval became the focus of our subsequent investigations (Figure 1).

### Alignment of Multiple MHC Haplotype Sequences Identifies 144 *PSORS1* Variants Associated with HLA-Cw*0602

As the latest version of dbSNP (build 137) lists 5,359 common variants (minor allele frequency >1%) mapping to the 179 kb *PSORS1* region, we sought to determine the number and distribution of changes unique to haplotypes bearing HLA-Cw*0602. By querying the GenBank database, we identified a number of publicly available MHC sequences which spanned the *PSORS1* locus and encompassed a variety of *HLA-C* alleles (Table 1). Since HLA-Cw*0602 was only found on three sequences, we sought to expand the catalogue of -Cw*0602 associated variants, by characterizing a fourth chromosome. We therefore sequenced the *PSORS1* region from the genome of a psoriatic individual who was heterozygous for HLA-Cw*0602. To circumvent the difficulty of mapping short genomic reads to a region as complex as the MHC, we generated a BAC library from the DNA of the patient and sequenced the two clones spanning the critical *PSORS1* interval (Figure S1 and Table S1).

**Table 1:** MHC haplotype sequences analysed in this study.

HLA-C allele	GenBank Accession n. (reference)
HLA-Cw*0602	KC312698 (this study)
HLA-Cw*0602	DQ249182 [20]
HLA-Cw*0602	DQ249178-DQ249180 [20]
HLA-Cw*0602	GL000252 [37]
HLA-Cw*030401	GL000254 [37]
HLA-Cw*030401	DQ249177 [20]
HLA-Cw*0501	GL000255 [37]
HLA-Cw*070101	GL000251 [37]
HLA-Cw*070101	DQ249172 [20]
HLA-Cw*070101	DQ249174 [20]
HLA-Cw*07020103	reference hg19 genome
HLA-Cw*07020103	DQ249181 [20]
HLA-Cw*0802	DQ249173 [20]
HLA-Cw*0802	DQ249176 [20]
HLA-Cw*1601	GL000253 [37]

A comparison of the sequences of the four -Cw*0602 bearing haplotypes with those of 11 chromosomes harbouring other *HLA-C* alleles (Table 1) indicated that only 151/5,359 *PSORS1* variants were unique to PSORS1-Cw*0602 haplotypes (i.e. they were present in at least three -Cw*0602 bearing sequences and absent from all other haplotypes). To further develop the panel of *PSORS1* chromosomes and increase the power to discriminate risk and non-risk variants, we next imputed *HLA-C* genotypes for 57 individuals of European descent, previously sequenced by the 1000 Genomes Consortium [12]. To take into account the relative uncertainty of MHC imputation data derived from low-coverage sequence reads, we defined as non-risk only those SNPs that were present in >10 non-Cw*0602 chromosomes. Through this approach we were able to exclude a further seven SNPs from our list of HLA-Cw*0602 associated alleles, bringing the total to count to one hundred and forty-four (Table S2).

**Figure 2:**
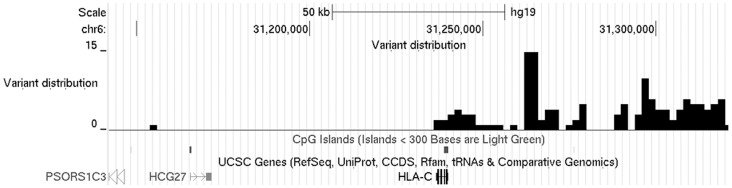
Distribution of *PSORS1* candidate variants. The number of changes that are unique to *PSORS1-Cw0602* haplotypes is plotted against GRCh37/hg19 coordinates.

The distribution of these candidate susceptibility variants was markedly skewed, with 111/144 markers located within a 72 kb region encompassing *HLA-C* (Figure 2). Of note, the only two nucleotide substitutions which mapped within the *HLA-C* coding region (rs1050414 and rs697743) were silent polymorphisms. Thus, neither of these variants is likely to have a pathogenic impact on protein function.

### A Molecular Characterization of the Refined *PSORS1* Interval Identifies Three Likely Enhancer Elements

We next sought to characterize the regulatory elements that map within the refined *PSORS1* locus. We initially queried the University of California Santa Cruz (UCSC) genome browser and assessed the position of open chromatin marks such as unmethylated CpG islands, DNAse hyper-sensitivity sites, histone H3 lysine 4 mono-methylation (H3K4me1) and histone H3 lysine 27 acetylation (H3K27ac). We focused our analysis on primary T lymphocytes, as these cells play a key role in the inflammatory responses underlying the pathogenesis of psoriasis [1]. We found that the refined *PSORS1* interval encompassed three DNA segments that presented multiple features associated with open chromatin (Table S3).

As the experimental data annotated in the UCSC genome browser had been generated in cells obtained from healthy individuals, we obtained blood samples from six psoriatic patients, in order to assess the distribution of key epigenetic marks in a context that was more relevant to the pathophysiology of the disease.

**Figure 3:**
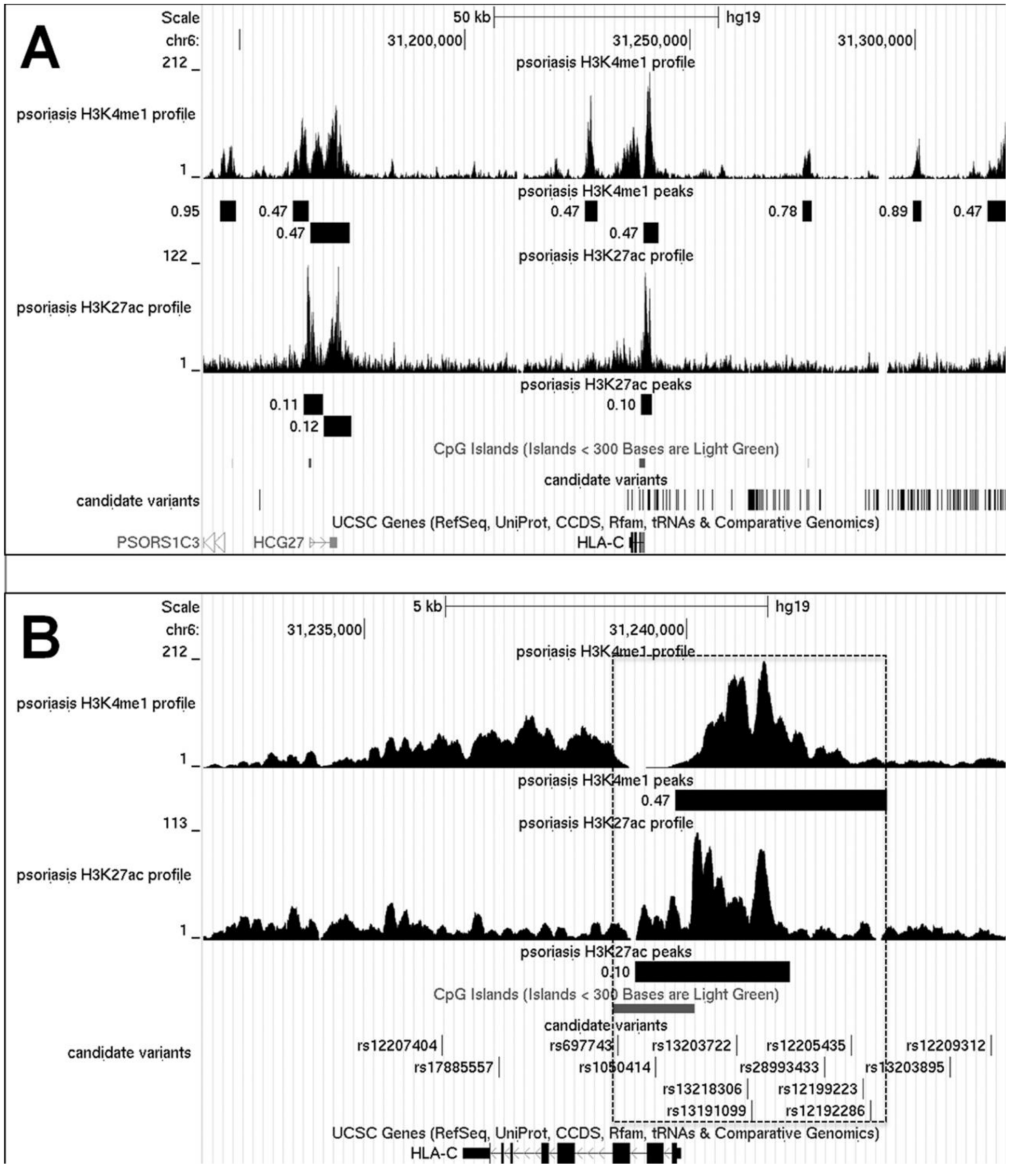
Epigenetic analysis of the refined *PSORS1* locus in patient T-lymphocytes. **A)** H3K4me1 and H3K27ac ChiP-Seq peaks are shown for a representative sample. The size of each peak is plotted as a black box immediately below the Chip-Seq track. The figure besides each box indicates the False Discovery Rate (FDR) associated with the identification of the peak (e.g. 0.47 indicates a FDR of 0.47%). **B)** A detailed view of the *HLA-C* gene region is shown. The dotted line box highlights the boundaries of the active regulatory region defined by the overlap of an unmethylated CpG island with H3K4me1 and H3K27ac peaks.

**Figure 4:**
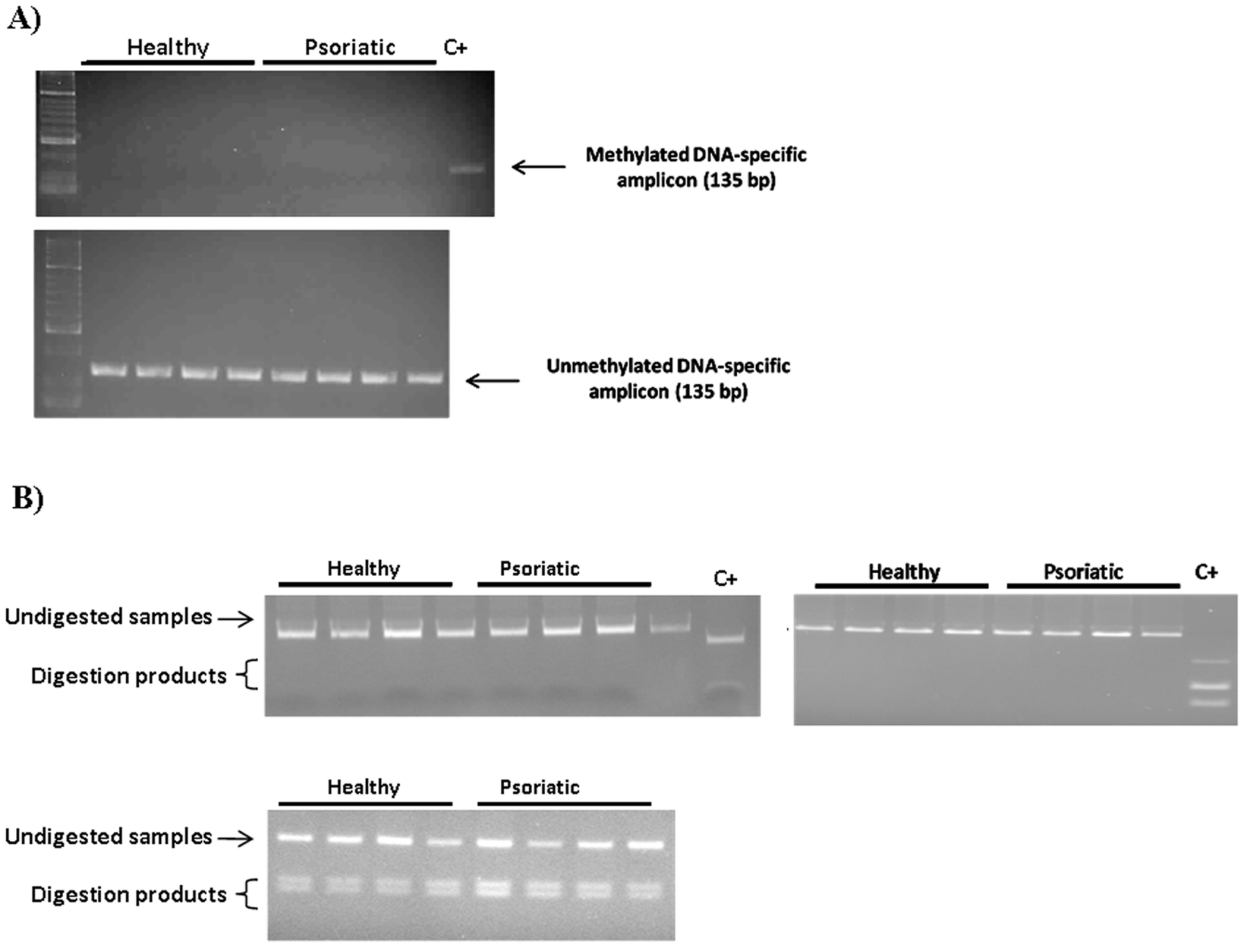
Locus specific analysis of whole-blood DNA identified similar methylation patterns in healthy and psoriatic individuals. (A) The CGI encompassing the minimal promoter and the first two exons *of HLA-C* was investigated by methyl-specific PCR, following treatment of whole blood DNA with sodium bisulfite. The DNA of a healthy donor was modified with the M.SssI CpG methyl-transferase and used as a positive control (C+). (B) The CGIs lying upstream of *HCG27* (top left), *HLA-C* (top right) and *POU5F1* (bottom) were analysed by CoBRA, exploiting the loss of restriction nuclease sites in unmethylated and bisulphite converted samples. A sample that did not undergo bisulfite treatment was used as positive control for the enzymatic digestion (C+). The methylation status of the CGIs was further validated by direct sequencing of cloned PCR products, generated from the DNA of one healthy and one affected individual (data not shown).

We first investigated the methylation status of the four CpG islands (CGIs) that lie within the refined *PSORS1* locus (shown in Figure 3A, immediately above the candidate variants track). We analysed the DNAs of four HLA-Cw*0602-positive patients and found that the CGI lying upstream of *POU5F1* was partially methylated, whereas the remaining three were completely unmethylated. This matched the modification pattern observed in control individuals (Figure 4).

We next investigated the distribution of H3K4me1 and H3K27ac binding sites, as these histone modifications are typically found together within active enhancers [13]. We used ChIP-Seq protocols to analyse DNA from the T-lymphocytes of two psoriatic patients who were heterozygous for HLA-Cw*0602 (Table S4). This identified nine H3K4me1 peaks, three of which overlapped with H3K27ac occupancy sites and are therefore likely to represent active enhancers. One of the three elements encompasses *HLA-C* exons 1–3, as well as the gene upstream region. The other two DNA segments map to a 10 kb region around the *HCG27* pseudogene (Figure 3). This pattern matches the distribution of H3K4me1 and H3K7ac sites observed in control T cells (Figure S2).

### Three Candidate Susceptibility Alleles Map to Biologically Relevant Transcription Factor Binding Sites within a Likely *HLA-C* Enhancer

We cross-referenced the position of all 144 SNPs unique to PSORS1-Cw*0602 haplotypes with that of the active regulatory elements identified by epigenetic profiling. While none of the variants map to the *HGC27* gene region, nine candidate susceptibility SNPs lie within the putative *HLA-C* enhancer element (Figure 3). To further explore the functional significance of these variants, we examined the data recently generated by the ENCODE Consortium [14]. We found that SNPs rs1050414 and rs697743 co-localize with experimentally validated binding sites for polymerase II and for POU2F2/Oct2, a well known immune activator [15]. Of note, rs1050414 also maps within a ChIP-Seq peak for NF-κB, a master regulator of inflammatory responses [16] (Figure S3).

To investigate the possibility that any of the nine critical SNPs may lead to the creation of novel transcription factor binding sites, we queried the TRANSFAC database using wild-type and variant sequences. This analysis indicated that SNP rs28993433 may introduce a novel site for SP4 (FDR <5%), a transcription factor involved in the regulation of MHC related genes [17].

## Discussion

The evidence supporting *PSORS1* as the major genetic determinant for psoriasis is overwhelming [2]. Of note, no secondary association signal has been detected within this susceptibility interval [18], [19], so that the disease risk conferred by *PSORS1* is likely due to a single genetic determinant. Although the results of GWAS and preliminary deep-sequencing studies point to HLA-Cw*0602 as a candidate susceptibility allele [6], [20], no aberrant function of the HLA-Cw6 protein has yet been demonstrated. Conversely, a number of studies have reported that HLA-C expression is altered in psoriatic lesions [5], [21] and that regulatory SNPs can affect *HLA-C* transcript levels [11], [22].

Here, we have undertaken a comprehensive molecular investigation of the *PSORS1* locus, with a view to dissecting the pathogenic contribution of SNPs that may affect the expression of HLA-Cw*0602.

We initially sought to refine the localization of the causal susceptibility allele(s), by analysing LD conservation patterns within the susceptibility interval. We exploited the unique dataset generated by the ImmunoChip Consortium, which includes 4,803 individuals, genotyped across the *PSORS1* locus at an average density of 1 SNP: 950 bp. The power of this approach is exemplified by the fact that it allowed us to refine the size of the susceptibility interval by nearly a third (from 259 to 179 kb), even though our assessment of LD conservation was rather conservative.

We next compared the sequence of HLA-Cw*0602 bearing chromosomes with that of publicly available non-risk haplotypes. This enabled us to generate a comprehensive catalogue of candidate susceptibility alleles and to exclude a pathogenic role for 97.3% of the common polymorphisms that lie within the refined *PSORS1* locus. Importantly, we showed that none of the 144 candidate variants resulted in an amino acid substitution that was unique to risk-bearing chromosomes. This observation argues against the possibility that the association at the *PSORS1* locus may be explained by a single amino acid change. Of note, recent studies have demonstrated that single non-synonymous substitutions account for the main MHC association signals underlying rheumatoid arthritis susceptibility and host control of HIV-I infection [23], [24]. Thus, our findings point to a potentially important difference in the make-up of the *PSORS1* allele and reinforce the notion that regulatory variants are likely to contribute to its pathogenic effect.

To investigate the biological significance of the 144 critical variants, we sought to identify functional non-coding elements mapping within the refined *PSORS1* interval. We initially queried the UCSC genome browser and mined data generated in T lymphocytes, as these cells drive key adaptive immune responses underlying the establishment of chronic inflammation in psoriasis [1]. We retrieved information on a variety of epigenetic marks and identified three likely active enhancers, mapping to the *HCG27* and *HLA-C* gene regions. Importantly, our in-house ChIP-Seq analysis confirmed that this epigenetic profile is also relevant to the transcriptional activity of patient T-lymphocytes. Our experiments were carried out in individuals who were heterozygous for HLA-Cw*0602, as the low frequency of the –Cw*0602/−Cw*0602 genotype hindered the recruitment of homozygous patients. Given the difficulties associated with the isolation of intact chromatin from skin keratinocytes or resident lymphocytes, the ChIP-Seq analysis was undertaken in circulating T-cells. Despite these limitations, the observation of comparable ChIP-Seq profiles in patients and controls indicates that the pathogenic effect of the *PSORS1* allele is unlikely to be mediated by the occurrence of major epigenetic alterations.

In the final phase of the study we integrated the results of our genetic and functional investigations and found that 9 candidate SNPs localize to a likely *HLA-C* enhancer. This element, which encompasses *HLA-C* exons 1–3 as well as the gene upstream region, spans approximately 5 kb and therefore extends significantly upstream of the canonical gene promoter identified by classic reporter studies [25]. The ChIP-Seq data generated by the ENCODE Consortium [14] indicate that this enhancer harbours binding sites for the NF-κB, STAT2, IRF4 and POU2F2 transcription factors, which all play important roles in inflammatory responses [15], [16], [26], [27]. Two of the *PSORS1* critical variants map within ChIP-Seq peaks for NF-κB and POU2F2, with a third predicted to create an SP4 binding site. While further studies will be required to validate the functional impact of individual SNPs, it is noteworthy that our data are entirely consistent with those generated in previous surveys of disease associate regions, which documented a highly significant enrichment of candidate susceptibility alleles within strong enhancer elements [13], [14].

In conclusion, we have carried out an in-depth molecular genetic investigation of the major psoriasis susceptibility locus and identified new regulatory variants that are likely to contribute to disease susceptibility. These findings pave the way for further immunological studies investigating the effects of altered HLA-Cw6 expression on inflammatory responses and psoriasis pathogenesis.

## Materials and Methods

### Ethics Statement

This study was conducted according to the principles expressed in the Declaration of Helsinki. All participating subjects granted their written informed consent. Ethical approval was obtained from Guy’s and St Thomas Hospital Local Research Ethic Committee.

### Subjects

The PSORS1-Cw*0602 sequence was generated from the DNA of an affected patient (JC), selected from an extended pedigree showing co-segregation of psoriasis with chromosome 6p21 markers. The analysis of epigenetic marks was undertaken in ten HLA-Cw*0602 heterozygous individuals (six patients and four controls), selected from a large case-control dataset that has been described elsewhere [6].

### High-resolution LD Conservation Analysis

The genotypes of 373 common SNPs (MAF >0.1) mapping to chr6∶31,062,133-31,419,877 were extracted from the data generated by the ImmunoChip consortium for 4,803 unrelated controls, selected from the 1958 British Birth Cohort and National Blood Service donors [3]. The –thin option of PLINK [28] was used to restrict the analysis to a random selection of 147 SNPs and a GOLD heatmap was generated using Haploview 4.2 [29]. The random selection process was repeated three times with comparable results.

### Generation of a PSORS1 HLA-Cw*0602 Sequence from Patient DNA

We generated a genomic BAC library using high molecular weight DNA, isolated from the immortalized lymphocytes of patient JC. We screened the library with a set of five probes, which matched unique *PSORS1* sequences within the *STG*, *CCHCR1, HCG27* and *HLA-C* gene regions. After validating the identity of eight positive clones by BAC end sequencing, we genotyped a set of informative SNPs located within the *CDSN, CCHCR1,* and *HLA-C* gene regions [30]. This allowed us to distinguish the clones bearing risk alleles from their wild type counterparts and led to the identification of two BACs carrying PSORS1-Cw*0602 sequences. These critical clones were sequenced by Cogenics (Grenoble, France) using a 454 Genome Sequencer FLX analyzer (Roche, Basel, Switzerland). A total of 125,011 reads were generated, 93% of which were mapped to the target sequence and processed with the analysis pipeline described in Figure S4. The resulting sequence was deposited in GenBank (accession n. KC312698).

### Identification of Variants Unique to PSORS1-Cw*0602 Haplotypes

Querying the GenBank database identified a total of 16 MHC haplotype sequences spanning the refined *PSORS1* locus. Of note, two of these included HLA-Cw*1203, a marker which has previously shown suggestive evidence for association with psoriasis [31], [32]. As the inconclusive nature of these findings prevented a clear definition of risk status for PSORS1-Cw*1203 chromosomes, these samples were excluded from all further analyses. We compared the sequences of the remaining *PSORS1* haplotypes using Sequencher v4.9 (Gene Codes Corporation, Ann Arbor, MI) to run the Clustal algorithm [33]. We identified 151 variants (excluding dinucleotide microsatellites and homopolymers exceeding 4 nucleotides in length) that were unique to PSORS1-Cw*0602 haplotypes. We next mined the data generated by the 1000 Genomes Project (available at ftp://ftp.1000genomes.ebi.ac.uk/vol1/ftp/) and used the genotypes of two critical SNPs (rs887466 and rs4379333 [24]) to predict the HLA-Cw*0602 status of 57 European individuals. We classified these individuals as HLA-Cw*0602 positive (n = 7), HLA-Cw*1203 positive (n = 4; removed from all further analyses) or HLA-Cw*0602/Cw*1203 negative (n = 46). Finally, we interrogated the genotypes of the HLA-Cw*0602/Cw*1203 negative individuals for each of the 151 variants identified in the previous phase. Variants found in >10 HLA-Cw*0602/Cw*1203 negative individuals were excluded from further analyses.

### Bioinformatic Analysis of the Refined *PSORS1* Interval

We queried the UCSC genome browser (http://genome.ucsc.edu/cgi-bin/hgGateway) for the annotation relating to the refined *PSORS1* locus (GRCh37/hg19 coordinates: 6∶31,142,245-31,321,211). We focused on the experimental data that had been generated in CD4^+^ T lymphocytes and collated information on open chromatin marks. We looked for DNase I hypersensitive sites (DHS) that were supported by a –log10(pval) >50, a signal value >30, and a score >120. We also annotated less significant peaks, which were found less than 1 kb away from any DHS site that met the above criteria. Finally, we scored the position of unmethylated CGI, H3K4me3 and H3K27ac sites.

We analysed the effect of SNPs that are unique to PSORS1-Cw*0602 and lie within regulatory elements, by querying the TRANSFAC database (BIOBASE Biological Databases, Beverly, MA) with the FIMO motif search tool, which is part of the MEME suite [34].

### DNA Methylation Analysis

Genomic DNA was treated with sodium bisulfite and purified using the EZ DNA Methylation-Gold™ Kit (Zymo, Orange, CA). The methylation status of the CGI encompassing the minimal promoter and the first two exons of *HLA-C* was investigated by methyl-specific PCR. Two independent reactions were carried out using either unmethylated DNA-specific (CpG121.UF1∶5′-GTGTTTTTTGGTTTTAATATTTTGG-3′; CpG121.UR1∶5′-CCACTTCATCTCAATAAACTACATA-3′) or methylated DNA-specific (CpG121.MF1∶5′-TTGTGTTTTTCGGTTTTAATATTTC-3′; CpG121.MR1∶5′-CGCTTCATCTCAATAAACTACGTA-3′) oligonucleotides. The methylation status of the remaining CGIs was investigated by combined bisulphite and restriction analysis (CoBRA). Sodium bisulphite treated DNA samples were amplified for 45 cycles with primers specific for each CpG islands (Table S5). Amplicons were digested using either TaiI or Taq^α^I (New England Biolabs, Ipswich, MA) and differentially methylated products were resolved on 3% agarose gels.

### ChIP-seq Analysis

Peripheral blood mononuclear cells were isolated from whole blood samples by centrifugation on a Ficoll (GE Healthcare, Little Chalfont, UK) layer and T cells were purified by negative selection, using the pan T cell Isolation Kit II Human, (Miltenyi Biotec, Bergisch Gladbach, Germany). Native chromatin was extracted from 25 million cells and fragmented by MNase enzymatic fragmentation. Immuno-precipitation was performed by incubating 3 µg of chromatin with 4 µg of anti H3K4me1 (pAb-037-050 from Diagenode, Liege, Belgium) or anti-H3K27ac (399133 from Active Motif, La Hulpe, Belgium) antibody. Following DNA recovery, ChIP-seq libraries were prepared and amplified (14 cycles) using the NEXTflex ChIP-seq kit from Bio Scientific (Austin, Texas, USA). Each sample was tagged using the NEXTflex ChIP-seq Barcodes. Libraries were normalized to 1 nM, pooled and sequenced on a HiSeq2000 apparatus (Illumina, San Diego, CA).

Pools were de-multiplexed with BCL (Illumina) and reads were mapped to the GRCh37/hg19 reference sequence using NovoAlign (Novocraft Technologies Sdn Bhd, Selangor, Malaysia). PCR duplicates were removed with MarkDuplicates (a utility available at http://picard.sourceforge.net/index.shtml) and poor quality alignments were removed with SAMtools [35]. ChIP-seq peaks were identified based on the depth of uniquely mapped reads, using MACS [36].

## Supporting Information

Figure S1 **Genomic location of the two BAC clones spanning the** ***PSORS1*** **locus.**
(DOCX)

Figure S2 **The** ***PSORS1*** **epigenetic profile of patient T-lymphocytes is comparable to that observed in controls.**
(DOCX)

Figure S3 **Candidate susceptibility SNPs co-localize with Pol-II, POU2F2 and NF-κB Chip-Seq peaks.**
(DOCX)

Figure S4 **Software pipeline used to analyze the sequence of the two BACs spanning the** ***PSORS1*** **locus.**
(DOC)

Table S1 **Sequence coverage statistics for the two BAC clones spanning the refined** ***PSORS1*** **locus.**
(XLSX)

Table S2 **Details of the 144 candidate** ***PSORS1*** **variants.**
(XLSX)

Table S3 **Distribution of** ***PSORS1*** **active chromatin marks in control T lymphocytes.**
(XLSX)

Table S4 **Sequence coverage statistics for the ChIP-Seq analysis of H3K4me1 and H3K27ac marks.**
(XLSX)

Table S5 **PCR primers and restriction enzymes used in the CoBRA analysis.**
(XLSX)
