# Supplementary material for: An In-Depth Characterization of the Major Psoriasis Susceptibility Locus Identifies Candidate Susceptibility Alleles within an HLA-C Enhancer Element
Source: PLoS One. 2013 Aug 19;8(8):e71690. doi: 10.1371/journal.pone.0071690 (PMC3747202; doi:10.1371/journal.pone.0071690)
Supplement: Figure S1 — Genomic location of the two BAC clones spanning the PSORS1 locus. (DOCX) [file pone.0071690.s001.docx]

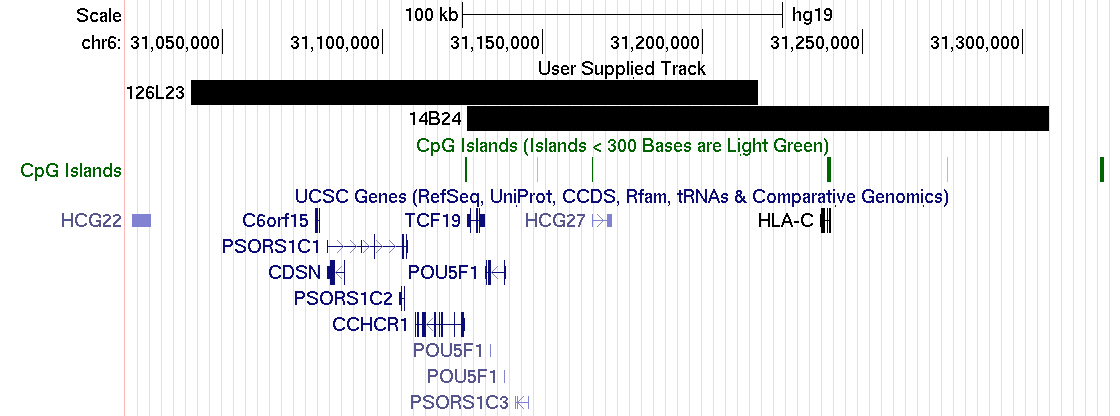


**Figure S1**. **Genomic location of the two BAC clones spanning the *PSORS1* locus.** Two BACs (each represented by a black rectangle) carrying *PSORS1* markers were isolated from a library covering the entire genome of an affected patient. The clones were mapped to GRCh37/hg19 coordinates (upper track), by means of BAC end-sequencing.
