# Supplementary material for: An In-Depth Characterization of the Major Psoriasis Susceptibility Locus Identifies Candidate Susceptibility Alleles within an HLA-C Enhancer Element
Source: PLoS One. 2013 Aug 19;8(8):e71690. doi: 10.1371/journal.pone.0071690 (PMC3747202; doi:10.1371/journal.pone.0071690)
Supplement: Figure S2 — The PSORS1 epigenetic profile of patient T-lymphocytes is comparable to that observed in controls. (DOCX) [file pone.0071690.s002.docx]

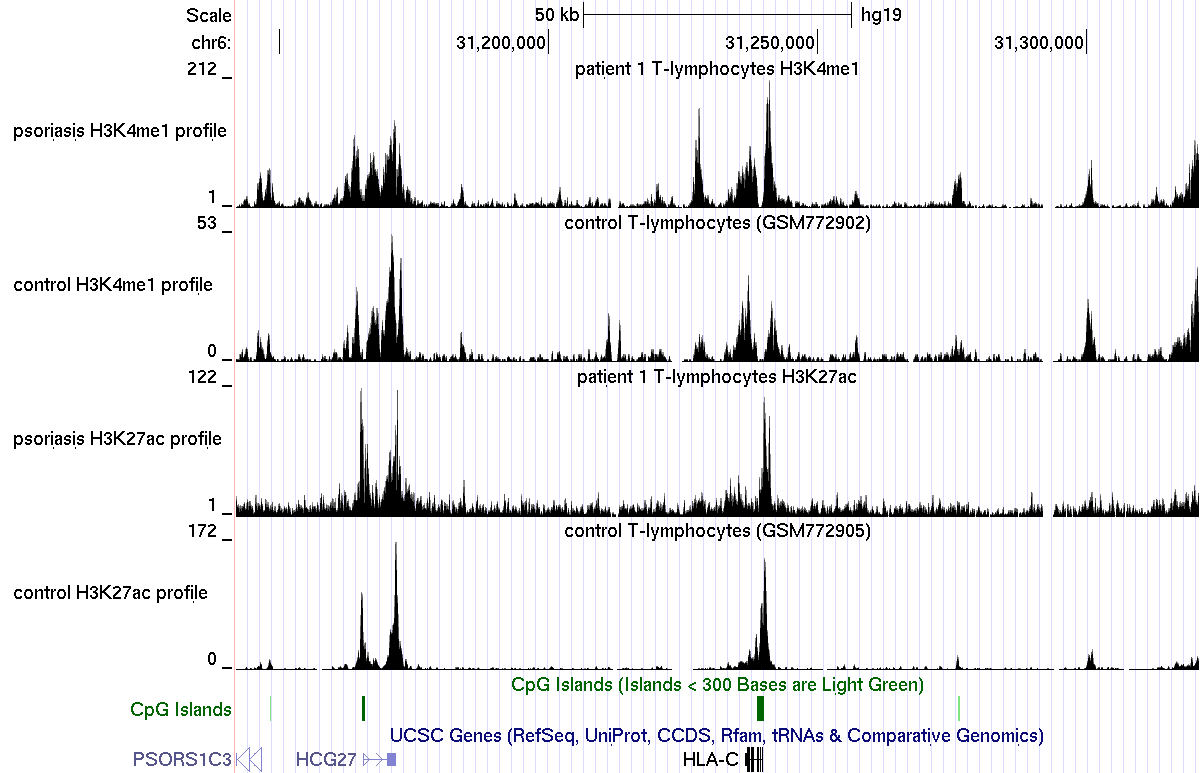


**Figure S2**. **The *PSORS1* epigenetic profile of patient T-lymphocytes is comparable to that observed in controls.** The H3K4me1 and H3K27ac sites identified in representative case and control samples are shown in the top four tracks, immediately below GRCh37/hg19 coordinates. The increased peak heights observed in patient DNA reflect the deeper coverage afforded by our HiSeq apparatus, compared to the GAII sequencer that was used to generate the publicly available control data.
