# Supplementary material for: An In-Depth Characterization of the Major Psoriasis Susceptibility Locus Identifies Candidate Susceptibility Alleles within an HLA-C Enhancer Element
Source: PLoS One. 2013 Aug 19;8(8):e71690. doi: 10.1371/journal.pone.0071690 (PMC3747202; doi:10.1371/journal.pone.0071690)
Supplement: Figure S3 — Candidate susceptibility SNPs co-localize with Pol-II, POU2F2 and NF-κB Chip-Seq peaks. (DOCX) [file pone.0071690.s003.docx]

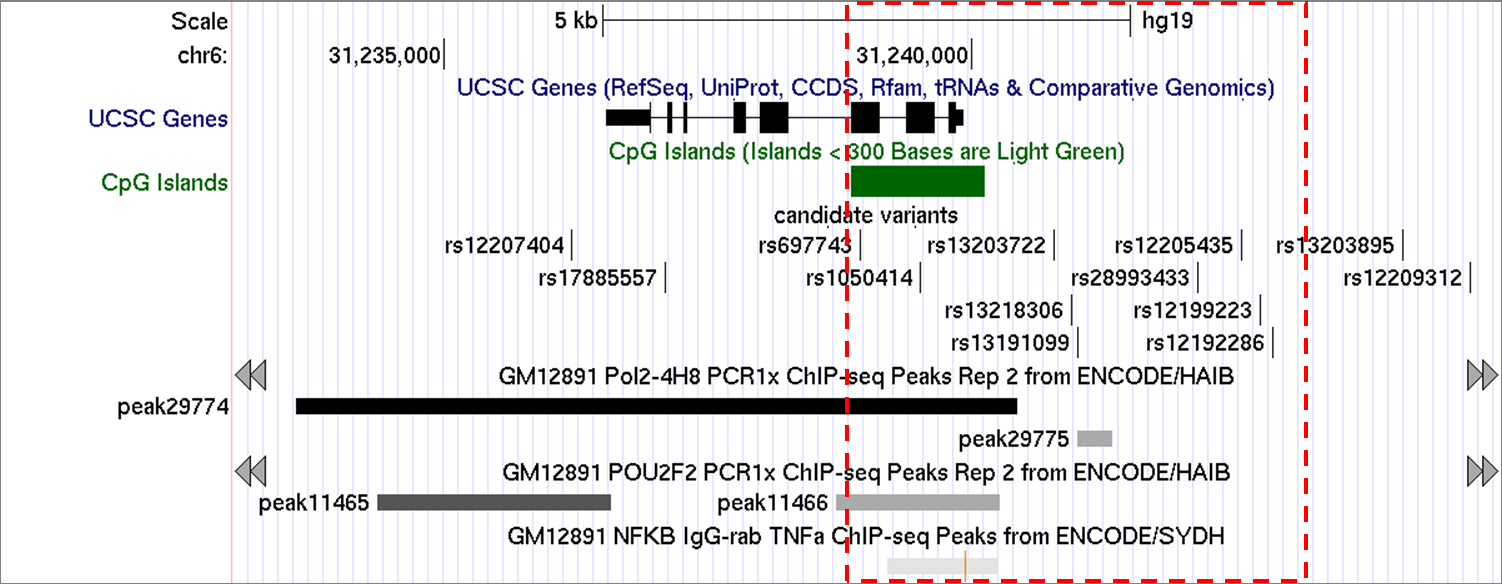


**Figure S3**. **Candidate susceptibility SNPs co-localize with Pol-II, POU2F2 and NF-κB Chip-Seq peaks.** A detailed view of the *HLA-C* gene region is shown. The red box highlights the boundaries of the active regulatory element, previously defined based on the overlap of an unmethylated CpG island with H3K4me1 and H3K27ac peaks. The position of ChIP-seq peaks indentified in the GM12891 lymphoblastoid cell line is shown by black and grey rectangles.
