# Supplementary material for: An In-Depth Characterization of the Major Psoriasis Susceptibility Locus Identifies Candidate Susceptibility Alleles within an HLA-C Enhancer Element
Source: PLoS One. 2013 Aug 19;8(8):e71690. doi: 10.1371/journal.pone.0071690 (PMC3747202; doi:10.1371/journal.pone.0071690)
Supplement: Figure S4 — Software pipeline used to analyze the sequence of the two BACs spanning the PSORS1 locus. (DOC) [file pone.0071690.s004.doc]

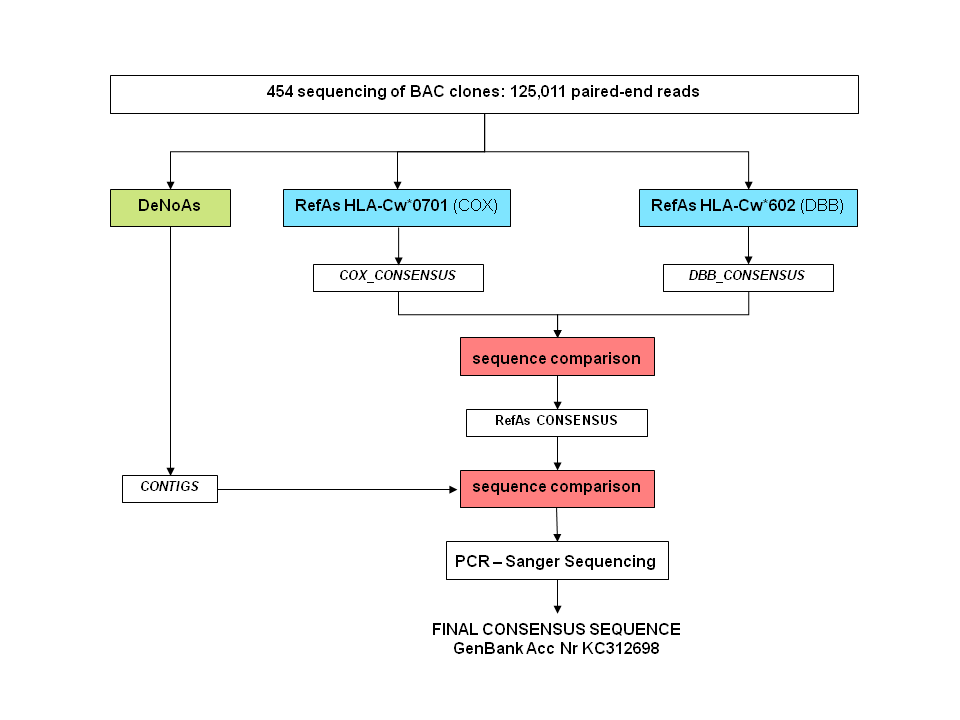
**Figure S4**. **Software pipeline used to analyze the sequence of the two BACs spanning the *PSORS1* locus.** To avoid any mapping bias, paired-end reads were aligned to two reference sequences. One was very similar to the target region *(PSORS1*-*Cw*0602),* while the second (*PSORS1*-*Cw*0701)* was expected to be significantly different. The two consensus sequences resulting from the alignment of the reads to *PSORS1*-*Cw*0602* (COX-CONSENSUS) and -*Cw*0701* (DBB_CONSENSUS) were then compared. Discrepancies were resolved by examining the sequence of contigs obtained by *de novo* assembly of the reads (DeNoAs). The discrepancies that could not be resolved *in-silico* were addressed by Sanger sequencing the BAC clone and the genomic DNA of the donor. Blue boxes refer to the alignments implemented by the CLC genomics workbench (CLC bio), whereas the green box denotes the *de novo* assembly generated by the Newbler software (Roche).
